# Romantic Relationship Length and its Perceived Quality: Mediating Role of Facebook-Related Conflict

**DOI:** 10.5964/ejop.v11i3.932

**Published:** 2015-08-20

**Authors:** H. M. Saidur Rahaman

**Affiliations:** a Department of Psychology, Jagannath University, Dhaka, Bangladesh; Aalborg University, Aalborg, Denmark

**Keywords:** Facebook, perceived relationship quality, Facebook-related conflict, relationship length

## Abstract

The purpose of this study was to investigate how Facebook use is leading to negative relationship outcomes such as cheating and breakup by assessing users’ perceived relationship qualities. It was hypothesized that Facebook-related conflict will be negatively related with users’ relationship length and will also be negatively related with their perceived relationship satisfaction, commitment, and love. Facebook-related conflict further mediates the relationship between relationship length and perceived relationship satisfaction, commitment, and love. Self-report data were gathered from participants (N = 101) in an online survey by employing standard questionnaires. A set of regression and mediation analyses confirmed all the hypotheses of the study. That is, Facebook-related conflict mediates the relationship between relationship length and perceived relationship satisfaction, commitment, and love. Moreover, the magnitude of mediation was highest for relationship satisfaction. Implications for future research and contributions are discussed.

Young people frequently use Facebook, which is often considered the most popular social network site (Duggan & Smith, 2013). Social scientists tend to focus on how Facebook is influencing our social life (see Wilson, Gosling, & Graham, 2012 for review). A recent study has showed that displays of emotion on Facebook contagiously influence others (Kramer, Guillory, & Hancock, 2014). This clearly indicates the extent of the influence Facebook has on people’s daily life. Excessive use of Facebook and Twitter can lead to negative relationship outcomes such as cheating, breakup, and divorce in the case of romantic relationship (Clayton, 2014; Clayton, Nagurney, & Smith, 2013). Social network sites (SNSs) use can negatively influence marriage quality and happiness and also positively influence the experience of troubled relationships and thought of separation (Valenzuela, Halpern, & Katz, 2014). From these findings, we may conclude that the impact of SNSs on an intimate relationship is pervasive, ranging from the pre-marital to the post-marital stage.

Though previous research identified the negative outcomes of SNSs use on intimate relationships, there is no study conducted yet that explores how these outcomes might emerge. The present research attempted to explore the relationship between Facebook use and negative relationship outcomes by assessing users’ perceived relationship qualities in the case of users who are currently involved in a romantic relationship. Practically, this study aims to contribute to developing strategies (e.g., couple therapy, awareness programs, campaigning etc.) to improve people’s quality of life in the face of new interpersonal communication platforms. Theoretically, it contributes to extending the implication of classical interpersonal communication theory and the relational turbulence model in the case of social networking and intimate relationships.

SNSs have a potential influence on intimate relationships at a personal or impersonal level. Social relationships demand interactions and SNSs are suitable platforms allowing this (Tong, 2013). A growing body of research has demonstrated that romantic relationships between partners can be in danger in the advent of partner’s Facebook jealousy, surveillance, ambiguous information presentation, compulsive Internet use, and online portrayals of intimate relationships (Kerkhof, Finkenauer, & Muusses, 2011; Muise, Christofides, & Desmarais, 2009; Papp, Danielewicz, & Cayemberg, 2012; Tokunaga, 2011; Utz & Beukeboom, 2011). Though SNS use might keep romantic partners in touch, its excessive use can be detrimental to the relationship (Clayton, 2014; Clayton et al., 2013; Joinson, 2008; Valenzuela et al., 2014).

Conflict may frequently occur in any intimate relationship (Brehm, Miller, Perlman, & Campbell, 2002). Clayton et al. (2013) introduced the construct ‘Facebook-related conflict’ in the context of intimate romantic relationship. They defined the construct ‘Facebook-related conflict’ as “whether Facebook use increases relationship complications in intimate romantic relationships” (p. 718). In their study, they found that the length of romantic relationship moderates the relation between Facebook use and Facebook-related conflict. And this conflict leads to negative relationship outcomes. This was particularly applicable for relationships that are lower in length and not fully matured. They pointed to the fact that perhaps due to lower relationship length, partners may keep track of their relational partners’ Facebook activities as an information-seeking technique for better knowing each other. However, the moderating role of relationship length was not found in another SNS (Twitter) use study (Clayton, 2014). This clearly indicates the inconsistent role of relationship length on Facebook-related conflict and demands further investigation. The accurate role of relationship length may not be explicated by treating it as a moderating variable but as an independent variable. Beside this, no study has been conducted yet to understand or explore how Facebook use relates to the perceived romantic relationship qualities of the users.

Uncertainty reduction theory (URT) posits that people seek information about their relational partners in order to reduce uncertainty concerning those partners and continue or pursue the relationship (Berger, 1979; Berger & Calabrese, 1975). The relational turbulence model further posits that relationships may experience different types of disruption even when the interaction between partners is working fine. At the beginning of any romantic relationship, partners want to know each other better. As the relationship grows mature and intimate, partners want to predict the future of the relationship. If they perceive any sort of relational uncertainty, they start to appraise one another negatively, experience jealousy and face difficulty in communicating with one another. If they cannot resolve that uncertainty, it is likely that they are going to end that relationship (Solomon & Knobloch, 2001; Solomon & Knobloch, 2004; Knobloch, 2007). Research demonstrates that jealousy is also a source of conflict in a romantic relationship (as cited in Fleischmann, Spitzberg, Andersen, & Roesch, 2005, p. 50). In this study I argue that, as the relationship grows, partners will be able to know each other better. Relational uncertainty will be reduced and the relationship will be stable with less occurrence of Facebook-related conflict.

Anderson and Emmers-Sommer (2006) define relationship satisfaction as “the degree to which an individual is content and satisfied with his or her relationship” (p. 155). Perceptions regarding various attitudes of the partner, partner’s behaviors, and communications between partners usually influence relationship satisfaction (Guerrero, 1994). Partner surveillance behavior and cognitive jealousy promote relationship dissatisfaction among undergraduate college students who are currently in a relationship (Elphinston & Noller, 2011). And disagreements over Facebook statuses with their romantic partners lower females’ relationship satisfaction (Papp et al., 2012). Cramer (2000) also found that conflict negatively correlates to relationship satisfaction. In line with these findings, I argue that Facebook-related conflict promotes relationship dissatisfaction.

According to Anderson and Emmers-Sommer (2006), commitment is “the extent to which people in romantic relationships experience relational cohesion (togetherness), exclusivity, and anticipated continuance of the relationship (dedication)” (p. 156). Studies showed that greater relationship commitment lowers the risk of infidelity (Drigotas, Safstrom, & Gentilia, 1999; McAnulty & Brineman, 2007). After starting any romantic relationship, partner’s Facebook solicitation behavior is the marker of lower relationship commitment (Drouin, Miller, & Dibble, 2014). Excessive Facebook use also promotes negative relationship outcomes (e.g., cheating, break up, divorce) via Facebook-related conflict (Clayton et al., 2013). These negative relationship outcomes are a sign of the partner’s lower level of commitment toward the relationship. It may be thus assumed that Facebook-related conflict lowers user’s relationship commitment.

The experience of love is central to any intimate relationship. A meta-analysis showed positive relationships between love and relationship satisfaction (Graham, 2011). Research also demonstrated that destructive conflict strategies negatively correlate to love (Zacchilli, Hendrick, & Hendrick, 2009). Thus, I argue that Facebook-related conflict will also be negatively correlated with user’s perceived love.

H1: Relationship length and Facebook-related conflict will be negatively related.

H2a: Facebook-related conflict will be negatively related with perceived relationship satisfaction.

H2b: Facebook-related conflict will be negatively related with perceived commitment.

H2c: Facebook-related conflict will be negatively related with perceived love.

Facebook-related conflict and Twitter-related conflict play a mediating role in predicting negative relationship outcomes (Clayton, 2014; Clayton et al., 2013). Combining *H1* and *H2*, I further argue that the lower the relationship length, the most Facebook-related conflict will occur. And this conflict will negatively influence partner’s perception of relationship satisfaction, commitment, and love.

H3a: Facebook-related conflict will mediate the relationship between relationship length and perceived relationship satisfaction.

H3b: Facebook-related conflict will mediate the relationship between relationship length and commitment.

H3c: Facebook-related conflict will mediate the relationship between relationship length and love.

## Methods

### Participants and Procedure

A total of 101 participants voluntarily responded to an online survey created for this study. A convenient sampling technique was used to recruit the respondents. At the beginning of the survey, a preface was included to obtain their consent, to provide information about the nature of the study, and to instruct them on how to fill up the survey form. Only participants who were currently in a heterosexual romantic relationship (not married) and in the relationship both partners used Facebook were asked to complete the survey. For having one or more important missing measures, five participants were excluded from the analysis. Among the participants, 61.46% were South Asian, 28.13% were European, and 10.41% were North American. The majority of them were female (51.04%). Their age ranged from 18 to 34 years (*M* = 24.73, *SD* = 3.32). All of the respondents were students (undergraduate, graduate, and Ph.D. researcher).

### Materials

Demographic questions (age, gender, and country of nationality), two standard survey questionnaires, and one single item questionnaire were used in the present study.

*Relationship Length*. Respondents were asked to report their relationship length in months in the online survey form. Their reported relationship length ranged from 3 to 120 months (*M* = 38.60, *SD* = 28.78).

The *Facebook-related Conflict Scale* (Clayton et al., 2013) is a six items questionnaire used to measure Facebook-related conflict (Cronbach’s α = .85). It measures how Facebook use may increase complications in an intimate relationship. Items include: “How often do you have an argument with your boyfriend/girlfriend as a result of excessive Facebook use?”, “How often do you have an argument with your boyfriend/ girlfriend as a result of viewing a friend’s Facebook profiles?”, “How often has Facebook led to a verbal dispute between you and your boyfriend/girlfriend (e.g., acceptance of a friend request, wall/picture comment, or a general post)?”, “How often do you feel jealous when other Facebook users comment on your boyfriend's /girlfriend's wall, photos, and statuses?”, “How often do you use Facebook to reconnect with individuals with whom you’ve had past romantic relationships?”, and “How often have you considered cheating on your boyfriend/ girlfriend with someone you have connected or reconnected with on Facebook?”. The Likert-type scale used ranged from ‘Never=0’ to ‘Always=5’. The Cronbach’s α for the scale in the present study was .76.

The *Perceived Relationship Quality Component (PRQC) scale* (Fletcher, Simpson, & Thomas, 2000) is a 18 items questionnaire consisting of six subscales which focus on measuring perceived relationship satisfaction, commitment, intimacy, trust, passion, and love of the individuals with regard to their romantic/intimate relationship quality. In the present study, only the relationship satisfaction, commitment, and love sub-scales were used. Each sub-scale consists of three items for which the Likert-type scaling ranged from ‘not at all=1’ to ‘extremely=7’. Sample items for relationship satisfaction included: “How satisfied are you with your relationship?”; for commitment: “How committed are you to your relationship?”; and for love: “How much do you love your partner?”. The Cronbach’s α for the scales used in the present study were: relationship satisfaction = .89, commitment = .84, and love = .79. The composite Cronbach’s α for the three factors of PRQC scale was .90.

*Control Variables.* Age, gender, and country of nationality were used in the analysis only as control variables.

*Confirmatory Factor Analyses.* To ensure that Facebook-related conflict, relationship satisfaction, commitment, and love were distinct constructs, confirmatory factor analyses were conducted. The result of one factor CFA revealed a poor fit (*χ^2^* = 336.85, *df* = 90, *p* < .001; CFI = .64; RMSEA = .17; SRMR = .12) to data. However, a four factor CFA solution revealed a far better model fit (*χ^2^* = 157.89, *df* = 80, *p* < .001; CFI = .89; RMSEA = .10; SRMR = .09). Thus, in testing hypotheses related to Facebook-related conflict, relationship satisfaction, commitment, and love, they were treated as distinct theoretical constructs.

## Results

Data obtained in the present study were analyzed by employing descriptive statistics, correlation, and mediation analysis. Table 1 presents the means, standard deviations, and correlations among the variables.

**Table 1:** Means, Standard Deviations, and Correlations for Variables

	*M*	*SD*	1	2	3	4	5
1. Relationship length	38.60	28.78	-	-.23*	.06	.16	.17
2. Facebook-related conflict	1.13	.91	-	-	-.37**	-.33**	-.36**
3. Relationship satisfaction	5.58	1.24	-	-	-	.53**	.58**
4. Commitment	5.80	1.15	-	-	-	-	.68**
5. Love	6.02	1.07	-	-	-	-	-

*Note.* *N* = 96.

*p < .05. **p < .01.

Regarding the variables investigated, the mean score of love was highest as one of the three components of perceived relationship quality. Correlation analysis showed that relationship length negatively and significantly correlates to Facebook-related conflict. Facebook-related conflict further significantly and negatively correlates to the three components of perceived relationship quality. Moreover, the three components themselves positively and significantly correlate with each other.

### Mediation Analyses

To test the mediating role of Facebook-related conflict, the conditional bootstrap analysis of Hayes (2013) was performed by employing his model 4 PROCESS macro for SPSS with 5000 bootstrap resamples. Though there was no significant direct relationship between the predictor and outcome variables in the present study, according to recent perspective and recommendation, there is still mediation to test (see MacKinnon, Fairchild, & Fritz, 2007; Rucker, Preacher, Tormala, & Petty, 2011; Shrout & Bolger, 2002 for review).

Firstly, relationship length was entered as an independent variable (*X*), relationship satisfaction (*Y*) as an outcome variable, and Facebook-related conflict (*M*) as a mediating variable in the model. A significant relationship was noted (*b* = -.0073, *p* < .05), demonstrating a negative relationship between relationship length and Facebook-related conflict [this supports H1]. A second significant relationship emerged (*b* = -.52, *p* < .001), demonstrating a negative relationship between Facebook-related conflict and relationship satisfaction [supporting H2a]. There was a significant indirect effect of Facebook-related conflict on relationship satisfaction through relationship length, *b* = .0038, 95% BCa CI [.0013, .0078]. By following the recommendation of Preacher and Kelley (2011) and guidelines of Cohen (1988), it appeared that κ^2^ = .09, 95% BCa CI [.0290, .1663] which indicates a medium level mediation effect. This supports H3a.

Secondly, relationship length (*X*) was entered as an independent variable, commitment (*Y*) as an outcome variable, and Facebook-related conflict (*M*) as a mediating variable in the model. A significant relationship emerged (*b* = -.0073, *p* < .05), demonstrating a negative relationship between relationship length and Facebook-related conflict [supported H1]. A second significant relationship emerged (*b* = -.40, *p* <.01), demonstrating a negative relationship between Facebook-related conflict and commitment [supported H2b]. There was a significant indirect effect of Facebook-related conflict on commitment through relationship length, *b* = .0029, 95% BCa CI [.0008, .0063]. By following the recommendation of Preacher and Kelley (2011) and guidelines of Cohen (1988), it appeared that κ^2^ = .07, 95% BCa CI [.0206, .1506] which indicates a near medium level mediation effect. This supports H3b.

Thirdly, relationship length (*X*) was entered as an independent variable, love (*Y*) as an outcome variable, and Facebook-related conflict (*M*) as a mediating variable in the model. A significant relationship emerged (*b* = -.0073, *p* < .05), demonstrating a negative relationship between relationship length and Facebook-related conflict [supporting H1]. A second significant relationship emerged (*b* = -.40, *p* < .01), demonstrating a negative relationship between Facebook-related conflict and love [supporting H2c]. There was a significant indirect effect of Facebook-related conflict on love through relationship length, *b* = .0029, 95% BCa CI [.0008, .0067]. By following the recommendation of Preacher and Kelley (2011) and guidelines of Cohen (1988), it appeared that *κ^2^* = .08, 95% BCa CI [.0230, .1628] which indicates a near medium level mediation effect. This supports H3c. In sum, all three proposed hypotheses in the present study were supported.

## Discussion

In this study, I explored how Facebook use is relating to negative relationship outcomes by assessing users’ perceived relationship satisfaction, commitment, and love. Results support all the proposed hypotheses. That is, users’ relationship length correlates negatively with Facebook-related conflict. Facebook-related conflict also correlations negatively with users’ perceived relationship satisfaction, commitment, and love. Furthermore, Facebook-related conflict mediates the relationship between relationship length and perceived relationship satisfaction, commitment, and love. Particularly, the magnitude of the mediation for relationship satisfaction appeared highest among the three.

The present findings make three main contributions. Firstly, the findings contribute to the growing body of literature pertaining to the domain of SNSs and their influence on intimate relationships by putting forward an explanation of how Facebook-related conflict is negatively related to relationship qualities. Secondly, this study contributes to the interpersonal communication and romantic relationship literature by suggesting the applicability of uncertainty reduction theory and relational turbulence model for explaining intimate relationships in the advent of new interpersonal communication platforms. More specifically, it explains the relationship among perceived relationship qualities, Facebook-related conflict, and relationship length using the theoretical framework of URT and relational turbulence model. Thirdly, it can contribute to developing relevant couple therapy and intervention techniques.

The present study sheds light on the findings of Clayton et al. (2013), Clayton (2014), and Valenzuela et al. (2014) as it brings evidence of the fact that Facebook use has the potential to decrease a user’s perceived relationship satisfaction, love, and commitment. This decrement of perceived relationship qualities may relate to negative relationship outcomes. The findings of the present are also consistent with the study findings of McDaniel and Coyne (2014) which demonstrate that technoference relates to conflict increment in relationship, and this conflict is partially responsible for the perception of dissatisfaction in the relationship.

Studies employing URT as a theoretical framework revealed that level of uncertainty in any intimate relationship substantially and inversely influences relationship stability (Knobloch & Solomon, 2002; Parks & Adelman, 1983); the continuity of any relationship will also be in danger if uncertainty cannot be reduced (Levine, Kim, & Ferrara, 2010). Relational turbulence model also suggests on how uncertainty in a romantic relationship may induce jealousy, communication difficulties, and partners negative appraisal toward one another (Knobloch, 2007; Solomon & Knobloch, 2001; Solomon & Knobloch, 2004). We can assume that Facebook use is causing conflict/argument between partners who are experiencing potential uncertainty regarding the stability or future of their relationship. Or, it induces the emergence of romantic jealousy between partners in case of a relatively new relationship that is still developing or maturing. This conflict eventually works towards lowering perceived relationship satisfaction. Partners may be inclined to explore relational alternatives or seek emotional support from readily available/approachable alternatives on Facebook, which indicates their decreased commitment towards the relationship. Conflict also lowers the magnitude of perceived love between partners. In sum, Facebook-related conflict negatively influences three aspects of the perceived quality of any romantic relationship. The extent of this negative influence depends on the length of that particular relationship. With increased relationship length, the Facebook-related conflict will decrease and the possibility of negative relationship outcomes will also be decreased.

On a practical note, it is important to understand how we can maintain a healthy relationship in the advent of new interpersonal communication platforms. The present study contributes in this regard by pointing to the relational factors which are potentially at risk because of SNSs use. Knowing these factors can help us develop relevant couple therapy or intervention technique and awareness programs (e.g., training, workshops) for maintaining a healthy romantic relationship. One practical suggestion for Facebook users, who are in a relatively new relationship, is that they should take part in open communication and honest discussion to reduce their relational conflict and uncertainty that may stem from Facebook-related conflict for maintaining an intimate and healthy relationship (McDaniel & Coyne, 2014; Theiss & Solomon, 2008).

### Limitations and Implications for Future Research

There are some limitations of the present study that should be mentioned. The cross-sectional nature of the data cannot capture how Facebook-related conflict actually develops over time. In this survey, it was not possible to rule out other general conflicts that may influence perceived relationship quality. As it was specified in the online survey, the study was meant to examine how Facebook use might influence relationship quality, so the data can be skewed due to desirability effects. Data in the present study were obtained from respondents who are currently in a romantic relationship but not married, divorced or separated. This specification significantly limits the generalization of the present study findings to broader populations. The sample included people living on different continents and future research can consider if there are any differences among them.

Future research should also investigate how Facebook-related conflict actually develops over time and how it influences perceived relationship quality by employing longitudinal designs. Experimental designs can allow us to study how SNSs (Facebook but also Twitter, Myspace, etc.) may influence, in causal terms, perceived relationship qualities. Other possible mediating variables can be used (e.g., mutual understanding between partners, loneliness, and trust) as they might influence relationship quality alongside Facebook-related conflict and this as well demands future research.

## Conclusion

This study explored the relation between Facebook use and negative relationship outcomes. Results indicate that users’ relationship length is negatively correlated with Facebook-related conflict and that Facebook-related conflict is also negatively correlated with perceived relationship satisfaction, love, and commitment. Facebook-related conflict further mediates the relationship between relationship length and perceived relationship satisfaction, commitment, and love. As such, although Facebook-related conflict negatively influences users’ perceived quality of romantic ties, the extent of these influences actually depends on the length of the relationship.
